# Capture vulnerability of sea turtles on recreational fishing piers

**DOI:** 10.1002/ece3.8473

**Published:** 2021-12-21

**Authors:** Margaret M. Lamont, Robert Mollenhauer, Allen M. Foley

**Affiliations:** ^1^ U.S. Geological Survey Wetland and Aquatic Research Center Gainesville Florida USA; ^2^ Florida Fish and Wildlife Conservation Commission Jacksonville Field Laboratory Fish and Wildlife Research Institute Jacksonville Florida USA

**Keywords:** boldness, capture vulnerability, Gulf of Mexico, pier, recreational fishing, sea turtle

## Abstract

Capture vulnerability of commercial and recreational fishes has been associated with behavioral, morphological, and life‐history traits; however, relationships with non‐target species, such as sea turtles, have not been adequately studied. We examined species composition, timing of captures, morphological variables including body size and head width, and body condition of sea turtles captured from a recreational fishing pier in the northern Gulf of Mexico and of sea turtles captured in the waters adjacent to the pier. From 2014 to 2019, 148 net captures and 112 pier captures of three sea turtle species were documented. Green turtles were captured most frequently in the net and on the pier. Turtles captured from the pier were larger than those captured in the net. There was no difference in head width between net‐caught and pier‐caught turtles; however, small sample sizes limited those comparisons. The body condition index was lower for pier‐caught than net‐caught Kemp’;s ridleys but did not differ with green turtles or loggerheads. Differences were also observed in the timing of capture on the pier as compared to in the net. Finally, the relationship between size, body condition, and pier‐capture vulnerability suggests these are complex interactions. Mortality of sea turtles captured from fishing piers could be selecting against bolder individuals, which may result in changes in sea turtle population demographics over a long time period.

## Graphical abstract

We examined species composition, timing of captures, morphological variables including body size and head width, and body condition of sea turtles captured from a recreational fishing pier in the northern Gulf of Mexico and of sea turtles captured in the waters adjacent to the pier. Green turtles were captured most frequently in the net and on the pier. Turtles captured from the pier were larger than those captured in the net.

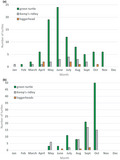

## 1 INTRODUCTION

Human population growth is increasing in coastal areas (Bounoua et al., 2018). This rapid rate of urbanization globally has led to dramatic changes to natural habitats (Gaston, 2010; Uchida et al., 2019). Consequently, some individual animals have adjusted their behaviors by foraging in new habitats or on novel prey items (Breck et al., 2019; Garamszegi et al., 2009; Tuomainen & Candolin, 2011). Drastic environmental changes often encourage individual behavioral traits such as boldness (Breck et al., 2019; Kelleher et al., 2017; Klefoth et al., 2017), which results in indirect selection against morphological and life‐history traits such as larger body size and faster growth rates (Alós et al., 2014; Enberg et al., 2012; Klefoth et al., 2017). These changes in individual traits can lead to increases in predation risk or vulnerability of capture in fishing activities of larger, bolder individuals (i.e., capture vulnerability; Klefoth et al., 2017; Phillip et al., 2009), thus ultimately leading to negative population‐level effects. As anthropogenic activities increase in coastal habitats, the response of wildlife, particularly imperiled species, could have far‐reaching consequences to ecology and conservation.

In the marine environment, one anthropogenic activity that has been shown to alter traits of target species is commercial and recreational fishing (Hamley, 1975; Klefoth et al., 2017; Lewin et al., 2006). In fact, commercial fishing is likely influencing the course of evolution for many target species (Enberg et al., 2012). Capture vulnerability has been shown to be a heritable trait in some fish species (Klefoth et al., 2017; Phillip et al., 2009), and behavioral traits such as boldness and morphological traits such as body size can impact catchability (Enberg et al., 2012; Uusi‐Heikkilä et al., 2008). Hook‐and‐line fishing, in particular, is more likely to capture individuals that are exploratory or have higher activity, boldness, or aggression levels. These behavioral traits increase encounters with fishing gear or increase the probability of ingesting certain baits or lures (Alós et al., 2012; Arlinghaus et al., 2016, 2017; Biro & Stamps, 2008; Diaz Pauli & Sih, 2017; Enberg et al., 2012; Lennox et al., 2017; Uusi‐Heikkilä et al., 2017).

Although increased capture vulnerability has been documented for many commercially and recreationally valuable fish species, many non‐target animals, including imperiled species such as sea turtles, are also often incidentally captured in fishing gear (Cook et al., 2020; Pate & Marshall, 2020). It would be expected that a suite of traits similar to those documented in fishes would increase sea turtle capture vulnerability. Sea turtles often forage in neritic waters (Bolten, 2003). Increases in incidental sea turtle captures by hook‐and‐line anglers, particularly from fishing piers, have recently been reported (Cook et al., 2020). Studies of sea turtles captured in recreational fishing activities have focused almost exclusively on individuals once they are captured (Cook et al., 2020; Rudloe & Rudloe, 2005; Seney, 2017) or after they have been released from subsequent rehabilitation (Coleman et al., 2016). Other studies have shown that juvenile green turtles (*Chelonia mydas*) exhibit individual differences in boldness (Griffin et al., 2017; Kudo et al., 2021). Capture vulnerability may also be affected by variations in the morphology, life‐history traits, and behavior of a species. Identifying such relationships may help managers design actions to reduce hook‐and‐line captures of protected species. For example, if a protected species co‐occurs with fishing activities during a certain time of the year, fishing activities could potentially be restricted at those times to reduce unintentional bycatch.

Globally, all sea turtle species, except the olive ridley (*Lepidochelys olivacea*), are listed as threatened or endangered by the U.S. Endangered Species Act and CITES. Five species of sea turtles are found in the Gulf of Mexico (GOM), including the Kemp’s ridley (*Lepidochelys kempii*), loggerhead (*Caretta caretta*), green turtle, leatherback (*Dermochelys coriacea*), and hawksbill (*Eretmochelys imbricata*) (Ward & Tunnell, 2017). The recovery plans for all of these species, except the leatherback (which is threatened by the pelagic longline fishery; Lewison et al., 2004), identify nearshore, recreational hook‐and‐line captures as a threat, and the Kemp’s ridley recovery plan specifically targets the reduction of hook‐and‐line interactions as a high‐priority action (National Marine Fisheries Service, 2011; National Marine Fisheries Service & U.S. Fish & Wildlife Service, 1991; National Marine Fisheries Service & U.S. Fish & Wildlife Service, 2008; Seminoff et al., 2015). Information on sea turtle bycatch and mortality in recreational fishing activities is minimal. We provide quantitative information on the vulnerability of sea turtles to recreational fishing, which can be used for developing management actions to reduce this source of mortality for sea turtle populations. The objectives of this project were to identify and compare characteristics that may affect capture vulnerability of sea turtles on a recreational fishing pier.

## 2 METHODS

**FIGURE 1:**
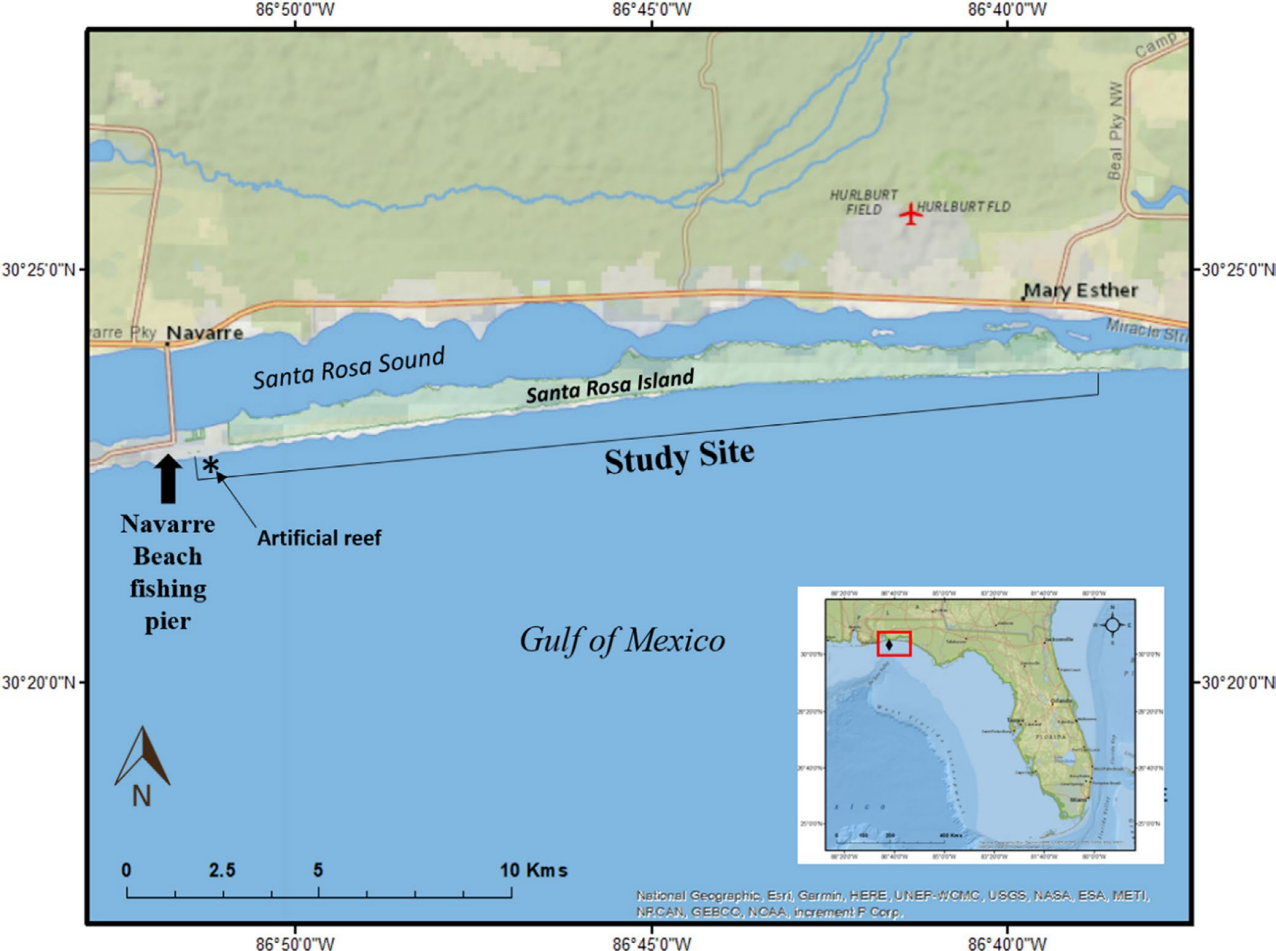
Locations where sea turtles were captured from 2014 to 2019 including the Navarre Beach fishing pier and surrounding waters off of Navarre Beach and Santa Rosa Island, Florida

We captured sea turtles along 21 km of Santa Rosa Island (SRI) owned by Eglin Air Force Base (AFB, Figure 1). The nearshore sediments in this area are predominately fine silica sand (Williams et al., 2012). The Navarre Beach (NAV) Marine Sanctuary, an artificial reef that consists of 78 structures constructed of piling‐mounted concrete disks located 340 feet offshore of the mean high tide line, lies approximately 0.5 km west of the study site. A fishing pier (NAV pier) is located on the GOM coast of SRI ~ 1 km west of the NAV Marine Sanctuary. This is the longest fishing pier along Florida’s GOM coast (Clark, 2010).

At the SRI study site, we captured turtles (hereafter net‐caught turtles) between March and November 2014–2019. Capture and sampling occurred following methods described by Lamont and Johnson (2021). Briefly, we surveyed for turtles from all‐terrain vehicles ridden on the beach. Once observed, we captured turtles using a modified set‐net technique in nearshore waters typically <2 m deep and within 100 m of shore. We marked all turtles with an Inconel tag in each front flipper and a passive integrated transponder (PIT) tag in one front flipper. We measured straight carapace length measured notch to tip (SCL) using metal calipers and curved carapace length measured notch to tip (CCL) using a cloth tape measure. Additionally, we used metal calipers to determine the straight head width following Price et al. (2017) and then calculated a relative head width (hereafter head width) by dividing head width by SCL. We determined weight (kg) by placing the turtle in a harness and hanging the harness from a hand‐held Pesola spring scale.

Data for turtles captured from the NAV pier (hereafter pier‐caught turtles) were collected by participants in the Florida Sea Turtle Stranding and Salvage Network using standardized protocols described by Foley et al. (2005). Pier‐caught individuals were taken to a local rehabilitation facility where they remained for varying lengths of time, depending on health status. The SCL, CCL, and weight were determined for pier‐caught turtles, but head width was not measured. Most individuals were released in nearby waters; however, some were relocated at distances >200 km.

Pairwise correlations (*r*) were .99 between weight, CCL, and SCL. We used SCL in all analyses. If SCL was not gathered for an individual, we converted CCL to SCL using the following regression equations from Teas (1993), where *r* ^2^ was >.95 for all modeled relationships:

(1) $$\text{SCL}=-1.\text{442C}\;\left(0.948\times \text{CCL}\right)\;\text{for loggerheads}$$

(2) $$\text{SCL}=-0.0\text{13C}\;\left(0.945\times \text{CCL}\right)\;\text{for Kemp}\text{’}\text{s ridleys}$$

(3) $$\text{SCL}=-0.294C\left(0.937\times \text{CCL}\right)\;\text{for green turtles}.$$

We calculated body condition index (BCI) as Fulton’s *K* (BCI = body mass/SCL^3^ × 10^4^; Bjorndal et al., 2000; Lamont & Johnson, 2021). The correlation between SCL and BCI was low (*r* = −.14).

### 2.1 Data analyses

#### 2.1.1 Body size and condition

**FIGURE 2:**
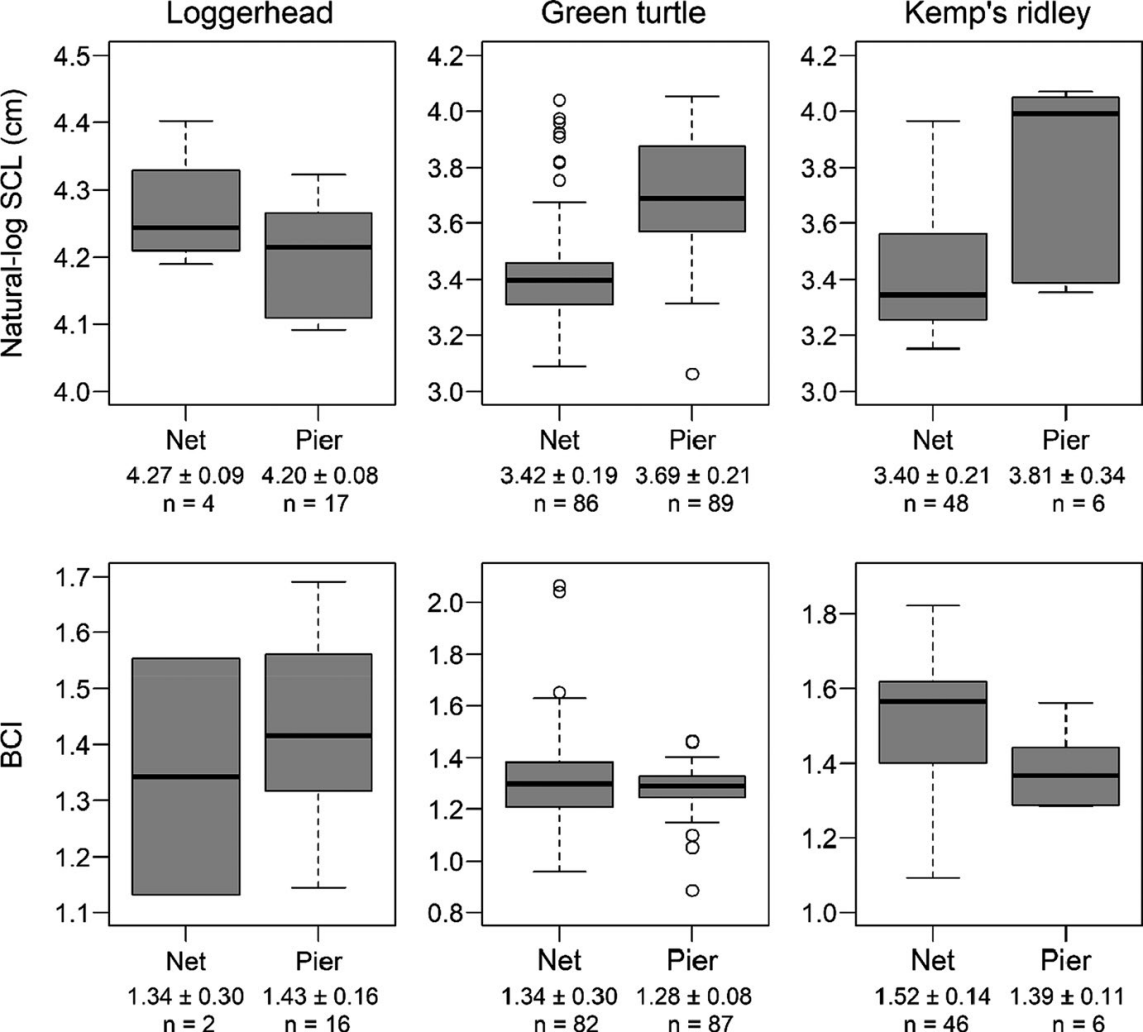
Summary statistics of pier‐caught and net‐caught sea turtles for straight carapace length (SCL) and body condition index (BCI). Values below the *x*‐axis labels are mean ± standard deviation (*SD*). Thicker vertical lines on the boxplots are medians. The number of observations (*n*) corresponds to the number of SCL and BCI measurements, where some turtles were observed more than once across time (see Methods). SCL is reported on a natural‐log scale to coincide with the log‐normal distribution used for the linear model. Mean ± *SD* in cm for net‐caught and pier‐caught turtles, respectively, was 71.73 ± 6.85 and 67.06 ± 5.52 for loggerhead, 31.06 ± 6.83 and 41.03 ± 8.30 for green turtle, and 30.79 ± 7.01 and 47.08 ± 14.07 for Kemp’s ridley

We used linear modeling to test for differences in SCL and BCI between pier‐caught and net‐caught sea turtles. We fit separate models with the same structure with SCL and BCI as the respective response variable *Y*. Each SCL and BCI observation was a measurement at the time of capture. Thus, turtles with more than one capture had multiple observations, each with unique measurements associated with either a net or pier capture. We could not directly account for individual fidelity in net or pier captures with SCL and BCL as *Y* because any lack of independence was at the observation, not turtle, level. Data plots supported a normal distribution assumption for BCI, but SCL was right‐skewed. Thus, we used a log‐normal distribution for SCL (see linear equations below). We treated species *j* as a factor with three levels (green turtle, Kemp’s ridley, and loggerhead) using a means parameterization (Gelman & Hill, 2007; Kéry & Royle, 2016). Pier caught or net caught was treated as an indicator (dummy) variable (pier caught = 0, net caught = 1). We accounted for variation in SCL and BCI due to time of year (hereafter day) using a covariate (i.e., continuous predictor variable). Day was quantified as an integer that coincided with the calendar day of observation *i* ranging from March 23 (day 82) to November 29 (day 334). We standardized day to a mean of zero and standard deviation (*SD*) of one. We also included a year *k* grouping factor (i.e., “random intercept”, Gelman & Hill, 2007) to account for unexplained annual variation in SCL and BCI. Standard deviations for both SCL and BCI were similar between pier‐caught and net‐caught turtles for each species (Figure 2). Thus, we assumed equal variances for both models. The linear equation can be written as:

(4) $$\begin{array}{c} Y_{\text{SCL}ijk}∼\text{Log - normal}\left(\mu_{\text{SCL}ijk},\sigma_{\text{SCL}}^{2}\right)\\ Y_{\text{BCI}ijk}∼N\left(\mu_{\text{BCI}ijk},\sigma_{\text{BCI}}^{2}\right)\\ \mu_{\mathrm{ijk}}=\alpha_{\mathrm{jk}}+\alpha_{1j}+\beta_{1j}{\text{Day}}_{i},\;\text{for}\;i=1,2\ldots N,\;\text{for}\;j=1,2\ldots J,\;\text{for}\;k=1,2\ldots K\\ \alpha_{\mathrm{jk}}∼N\left(0,\sigma_{\alpha jk}^{2}\right), \end{array}$$

where *α* is pier‐caught, *α*_1_ is net‐caught, *β*_1_ is the day slope, *µ* is mean, and *σ* ^2^ is variance. We report SCL and BCI relationships for each species as the mode (effect size) of the difference between pier‐caught and net‐caught turtles with a 90% highest density interval (HDI). Differences were considered significant if the HDI did not overlap zero (Kruschke & Liddell, 2019). The complete linear model estimates are included in Table 1. We assessed model fit using a posterior predictive check of observed versus simulated *Y* values (Kéry & Royle, 2016). In addition to a visual examination, we formally assessed fit using a Bayesian *p*‐value. A Bayesian *p*‐value .10–.90 supports adequate fit (Conn et al., 2018; Kéry & Royle, 2016).

**TABLE 1:** Linear model estimates for straight carapace length (SCL) and body condition index (BCI) used for the pier‐caught and net‐caught multiple comparisons tests (Table 1)

Parameter	SCL	BCI
Green turtle—pier	3.43 (3.34, 3.52)	1.31 (1.28, 1.35)
Green turtle—net	−0.20 (−0.27, −0.14)	−0.03 (−0.08, 0.01)
Green turtle—day	0.01 (−0.02, 0.04)	−0.01 (−0.04, 0.01)
Kemp’s ridley—pier	3.36 (3.26, 3.46)	1.52 (1.48, 1.56)
Kemp’s ridley—net	−0.53 (−0.68, −0.38)	−0.11 (−0.22, −0.01)
Kemp’s ridley—day	0.11 (0.05, 0.17)	−0.01 (−0.06, 0.03)
Loggerhead—pier	4.22 (4.03, 4.41)	1.40 (1.24, 1.53)
Loggerhead—net	0.00 (−0.18, 0.19)	0.02 (−0.12, 0.18)
Loggerhead—day	0.04 (−0.03, 0.11)	−0.01 (−0.10, 0.03)

Note

Coefficients are reported on the natural‐log and raw scale for SCL and BCI, respectively, as the mode (effect size) with a 90% highest density interval (HDI). Net is interpreted as the difference between net‐caught turtles and pier‐caught turtles at mean time of year (day). Year standard deviation (*SD*) represents unexplained annual variation in SCL and BCI across all species.

We fit the models using the program JAGS (Plummer, 2003) called from the package jagsUI (Kellner, 2018) within the statistical software R (version 3.5.3; R Development Team, 2019). Posterior distributions for coefficients were estimated with Markov chain Monte Carlo (MCMC) methods using two chains of 15,000 iterations each after a 5000‐iteration burn‐in phase (no thinning). We assessed model convergence using the Brooks–Gelman–Rubin statistic ($\hat{R}$, Gelman & Rubin, 1992). $\hat{R}$ < 1.1 for all parameters indicates adequate chain mixing (Kruschke, 2015). We also examined parameter trace plots to confirm good convergence.

#### 2.1.2 Head width and body condition

**FIGURE 3:**
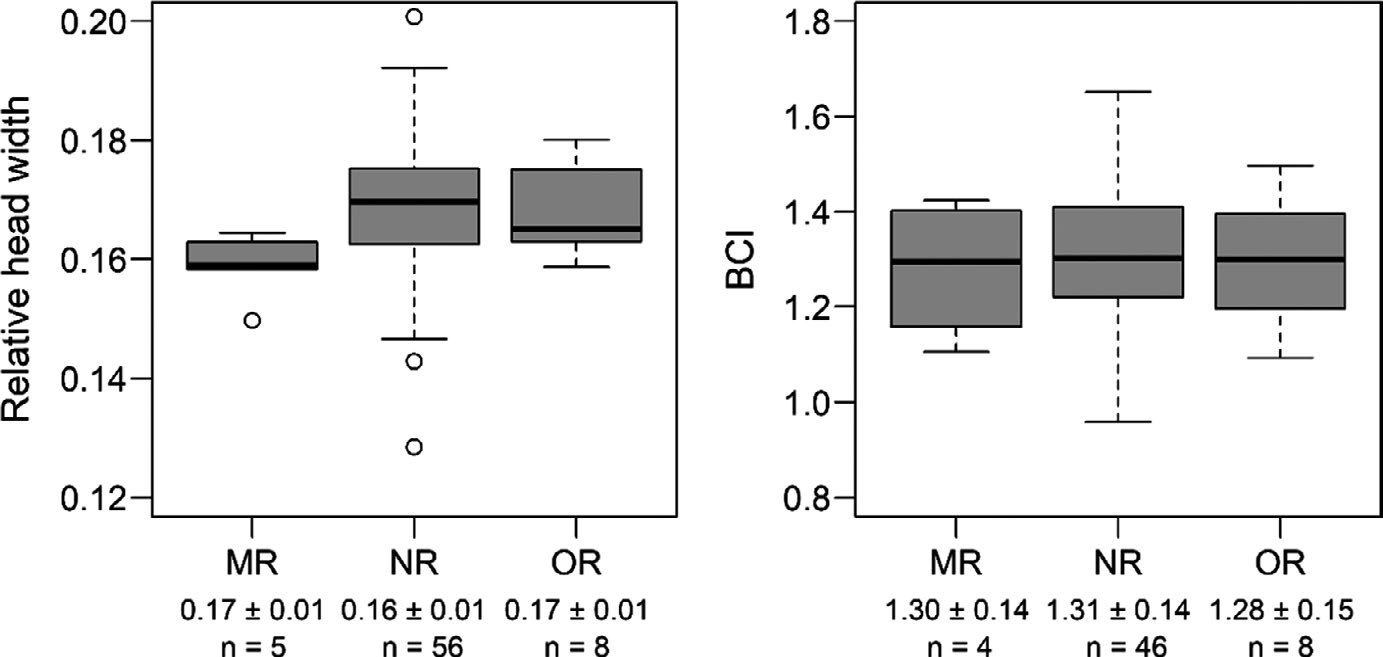
Summary statistics of relative head width and body condition index (BCI) for net‐caught green turtles with different numbers of pier captures. MR is multiple pier captures, NR is no pier captures, and OR is one pier capture. Values below the *x*‐axis labels are mean ± standard deviation (*SD*). Thicker vertical lines on the boxplots are medians. The number of observations (*n*) corresponds to the number of turtles, where only one relative head width and BCI value was assigned to each (see Methods)

We used the same general model structure as the pier‐caught and net‐caught SCL and BCI comparisons to test for differences in head width and body condition between groups of green turtles with different numbers of pier captures. Because head width was only determined for turtles that were net‐captured, we could only use pier‐captured turtles that were initially captured by net. For turtles that were captured multiple times by net, we used the mean for head width. Loggerheads and Kemp’s ridleys were not included in this analysis because head width was not measured on the majority of individuals. We fit identical models treating head width and BCI as the respective *Y* variable. A plot of head width supported a normal distribution assumption. A recapture factor comprised three levels associated with each net‐caught green turtle: no pier captures (i.e., only net caught), one pier recapture of a net‐caught individual, and multiple pier recaptures of net‐caught individuals. Standard deviations for both head width and BCI were similar among the recapture levels (Figure 3). We used multiple comparisons (Kruschke, 2015; Kruschke & Liddell, 2019) to test for differences in green turtle head width and BCI between the following groups of net‐caught turtles: no pier captures versus one or multiple pier captures, no pier captures versus one pier capture, no pier captures versus multiple pier captures, and one pier capture versus multiple pier captures. The complete linear model estimates are included in Table 2. We fit models using JAGS with the same MCMC settings and assessed significance, fit, and model convergence as described for the pier‐caught and net‐caught SCL and BCI comparisons.

**TABLE 2:** Linear model estimates for green turtle relative head width (head width) and body condition index (BCI) used for the multiple comparisons tests (Table 2)

Parameter	Head width	BCI
No pier captures	0.17 (0.17, 0.17)	1.31 (1.28, 1.35)
One pier capture	0.17 (0.16, 0.17)	1.30 (1.21, 1.38)
Multiple pier captures	0.16 (0.15, 0.17)	1.28 (1.16, 1.40)
*SD*	0.01 (0.01, 0.01)	0.15 (0.12, 0.17)

Note

Coefficients are reported on the raw scale as the mode (effect size) with a 90% highest density interval (HDI). *SD* is standard deviation.

#### 2.1.3 Pier‐capture vulnerability

We examined green turtle pier‐capture vulnerability in relation to SCL, BCI, and day. Day provided a surrogate for both general tendencies of being pier caught at certain times of year (e.g., increased boldness) and seasonal variation in fishing effort. We did not include loggerhead or Kemp’s ridley in this analysis due to the small number of captures from the pier. Pier‐capture vulnerability Ψ was treated as a Bernoulli process (*Y* = 0 if net caught, *Y* = 1 if pier caught) and modeled as a linear function of covariates. Straight carapace length was natural‐log transformed due to a right‐skewed distribution. All covariates were standardized to a mean of zero and *SD* of one. We also included an individual *s* grouping factor to account for pseudoreplication and unexplained individual variation associated with repeat measurements of individuals (Wagner et al., 2006). The linear model can be written as:

(5) $$\begin{array}{c} Y_{\mathrm{is}}∼\text{Bernoulli}\left(\Psi_{\mathrm{is}}\right)\\ \text{logit}\left(\Psi_{\mathrm{is}}\right)=\alpha_{s}+\beta_{1}{\text{SCL}}_{i}+\beta_{2}{\text{BCI}}_{i}+\beta_{3}{\text{Day}}_{i},\;\text{for}\;i=1,2,\ldots N,\;\text{for}\;s=1,2,\ldots S,\\ \alpha_{s}∼N\left(0,\sigma_{\alpha s}^{2}\right) \end{array}$$

We considered a covariate significant if the 90% HDI for the slope did not overlap zero. We fit the model using JAGS with MCMC settings as four chains of 50,000 iterations each after a 25,000‐iteration burn‐in phase (thinning = 10). We assessed model convergence using $\hat{R}$ and parameter trace plots.

## 3 RESULTS

### 3.1 Net‐caught turtles

**FIGURE 4:**
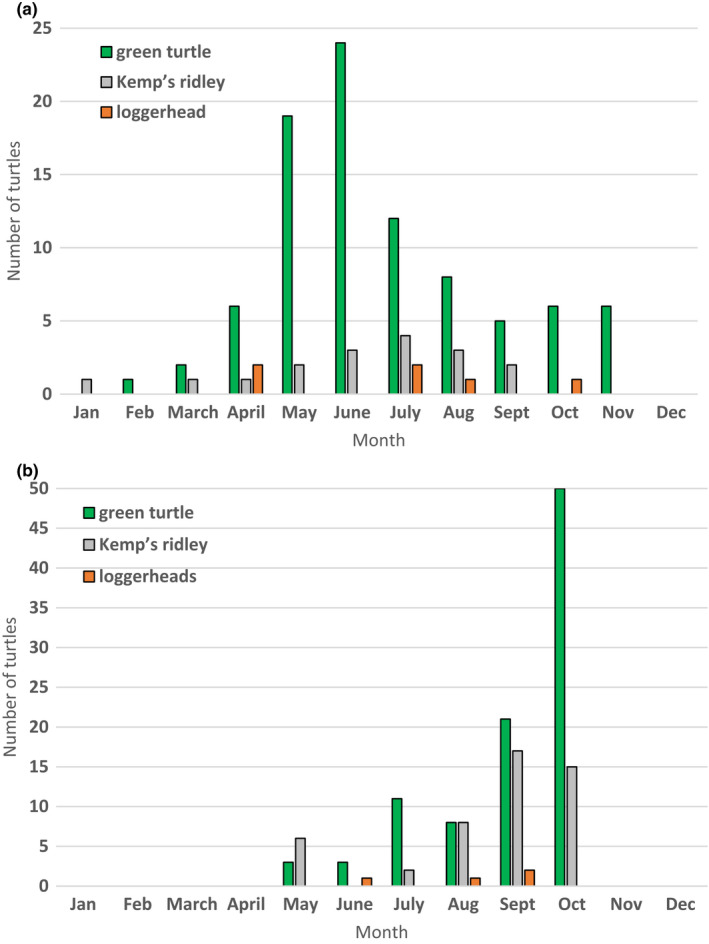
Number of turtles by species captured per month on the Navarre Beach fishing pier (a) and by net off of Santa Rosa Island, Florida (b)

From October 2014 to October 2019, 148 captures of 102 unique individuals occurred in the nearshore waters at our study site (Figure 2). Captures were attempted in every month from May to October. Sampling effort was greatest in September (27% of sampling days), but most turtles were captured in October (44%; Figure 4). Green turtles were caught most frequently followed by Kemp’s ridleys and loggerheads.

Twelve net‐caught turtles were also caught (either before or after) on the pier. Our overall recapture rate (i.e., either net or pier recaptures) of net‐caught turtles was 29%. Most recaptured turtles (67%) were only recaptured once, 30% were recaptured twice, and 4% were recaptured three times. We recaptured green turtles more frequently (72%) than Kemp’s ridleys (28%); we did not recapture any loggerheads. Mean recapture interval was 111 d and was longer for Kemp’s ridleys (185 d; range 12–366 d) than green turtles (72 days; range 1–391 d). Of turtles recaptured in the net, 12 were originally tagged after being caught by hook and line on the NAV pier (27%). Mean weight ± *SD* for net‐caught turtles was 5.34 ± 6.39 kg (range: 1.40–50.40).

### 3.2 Pier‐caught turtles

From 2014 to 2019, 112 captures of 78 unique individuals were captured by hook‐and‐line on the NAV pier (Figure 2). Captures occurred in every month except December; most (24%) occurred in June. On the pier, green turtles were captured most frequently followed by loggerheads and Kemp’s ridleys. Green turtles were captured most frequently in June (27%) and May (21%), whereas Kemp’s ridleys were captured most frequently in April (33%) and July (33%) and loggerheads in July (23%; Figure 4). Most (58%) pier‐caught turtles were foul‐hooked, whereas 30% were hooked in the mouth, and the remaining 12% were hooked in unreported locations. Hooking locations were similar among species with the fewest mouth‐hooked individuals reported in loggerheads (24%) as compared to green turtles (30%) and Kemp’s ridleys (33%). Mean weight ± *SD* for pier‐caught turtles was 15.04 ± 12.89 kg (range: 1.30–56.90).

Of all pier‐caught turtles, 30% were already tagged (i.e., they were recaptures). Of those individuals, 14% (*n* = 16) were originally net caught and then recaptured from the pier; mean recapture interval for those individuals (i.e., net caught to pier caught) was 832 days (*SD* = 487.8, range 57–1422 days). Of all turtles tagged after being pier caught, most were captured only once (55%), whereas 30% were captured twice, 10% were captured three times, and one individual (5%) was captured 5 times during the study period. Of all pier recaptures (individuals captured at least twice from the pier), most (85%) were green turtles followed by loggerheads (16%); no Kemp’s ridleys were recaptured from the pier. Mean pier‐recapture interval for all turtles was 333 d and was longer for green turtles (347 d; range 26–1422 d) than for loggerheads (266 d; range 42–473 d).

### 3.3 Data analyses

#### 3.3.1 Pier‐caught versus net‐caught size and body condition

Both SCL and BCI differed significantly between pier‐caught and net‐caught turtles for at least one species (Table 3). Straight carapace length was significantly greater for both pier‐caught green turtles and Kemp’s ridleys, with a larger effect size for Kemp’s ridley (Table 3). In contrast, BCI was significantly lower for pier‐caught Kemp’s ridley. Neither SCL nor BCI differed significantly for loggerheads, with a small effect size for both relationships. Diagnostic plots indicated good fit, with a Bayesian *p*‐value of .51 and .49 for the SCL and BCI model, respectively. $\hat{R}$ was <1.05 for all model parameters, and trace plots confirmed adequate convergence.

**TABLE 3:** Comparisons of straight carapace length (SCL) and body condition index (BCI) between pier‐caught (pier) and net‐caught individuals (net) for green turtle, Kemp’s ridley, and loggerhead (also see Table 1)

Comparison	SCL (cm)	BCI
Green turtle—pier versus net	*6.94 (4.77, 9.22)	−0.03 (−0.08, 0.01)
Kemp’s ridley—pier versus net	*20.10 (13.00, 27.68)	*−0.11 (−0.22, −0.01)
Loggerhead—pier versus net	0.21 (−12.43, 12.79)	0.02 (−0.12, 0.18)

Note

Coefficients for SCL and BCI reported as the mode (effect size) of the difference with a 90% highest density interval (HDI). The direction of the tests is left minus right. *Highlights HDIs that did not overlap zero. For example, the finding for “green turtle—pier versus net” is that mean SCL was greater for pier‐caught green turtles than net caught, and the difference was significant. Mean BCI was greater for net‐caught green turtles; there it was not significant difference from pier‐caught turtles.

#### 3.3.2 Pier recaptures head width and body condition

There were no significant differences in either head width or BCI among green turtles with different numbers of pier recaptures (Table 4). Diagnostic plots indicated good fit, with a Bayesian *p*‐value of .53 and .50 for the head width and BCI model, respectively. $\hat{R}$ was <1.05 for all model parameters, and trace plots confirmed adequate convergence.

**TABLE 4:** Multiple comparisons of relative head width (head width) and body condition index (BCI) between groups of net‐caught green turtles with different numbers of pier captures (also see Table 2)

Comparison	Head width	BCI
No pier captures versus one or multiple pier captures	0.01 (−0.01, 0.01)	0.02 (−0.06, 0.11)
No pier captures versus one pier capture	0.00 (−0.01, 0.01)	0.02 (−0.07, 0.11)
No pier captures versus multiple pier captures	0.01 (−0.01, 0.02)	0.03 (−0.09, 0.16)
One pier captures versus multiple pier captures	0.01 (−0.01, 0.02)	0.02 (−0.13, 0.16)

Note

Coefficients are reported on the raw scale as the mode (effect size) with a 90% highest density interval (HDI). The direction of the tests is left minus right.

#### 3.3.3 Pier‐capture vulnerability

**FIGURE 5:**
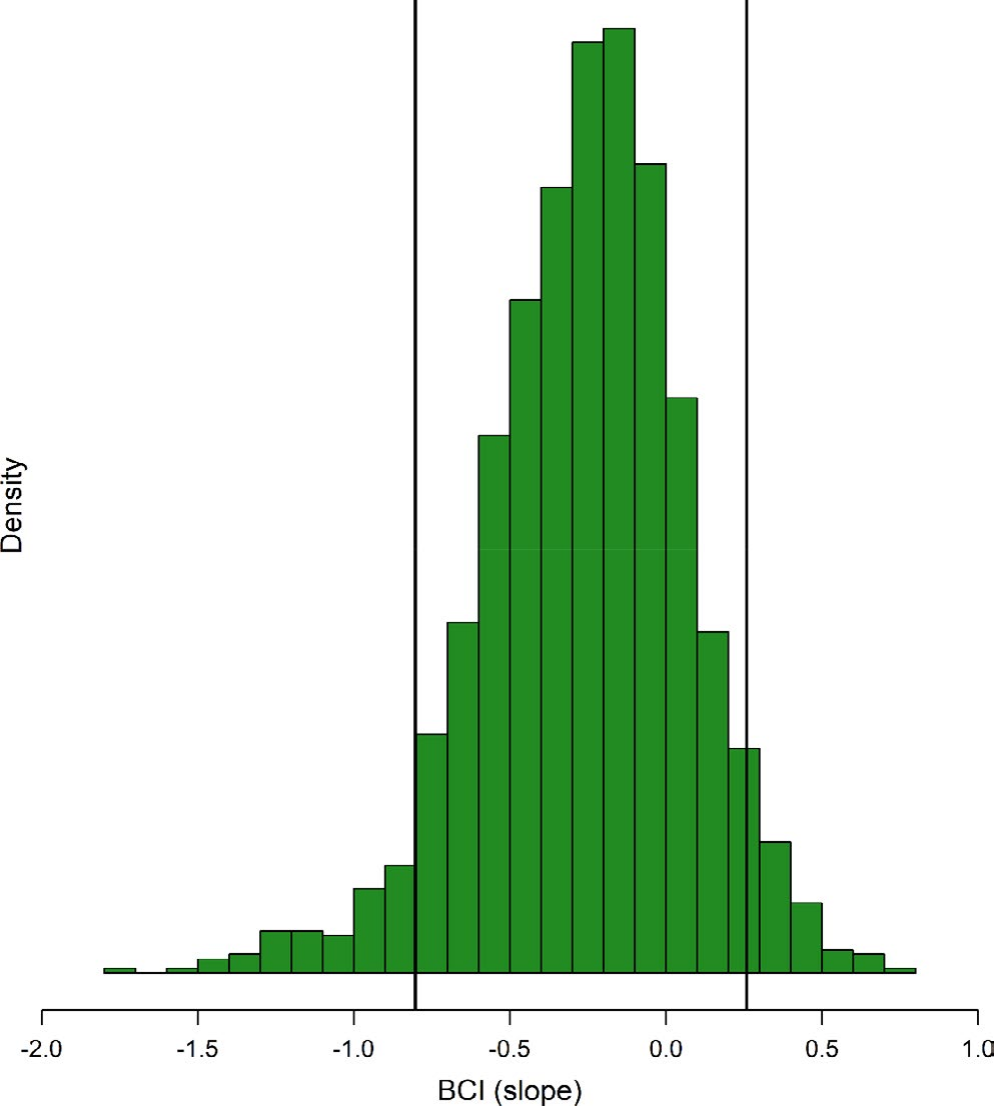
Posterior distribution of the body condition index (BCI) slope with a 90% highest density interval vertical lines from the green turtle pier‐use model

**FIGURE 6:**
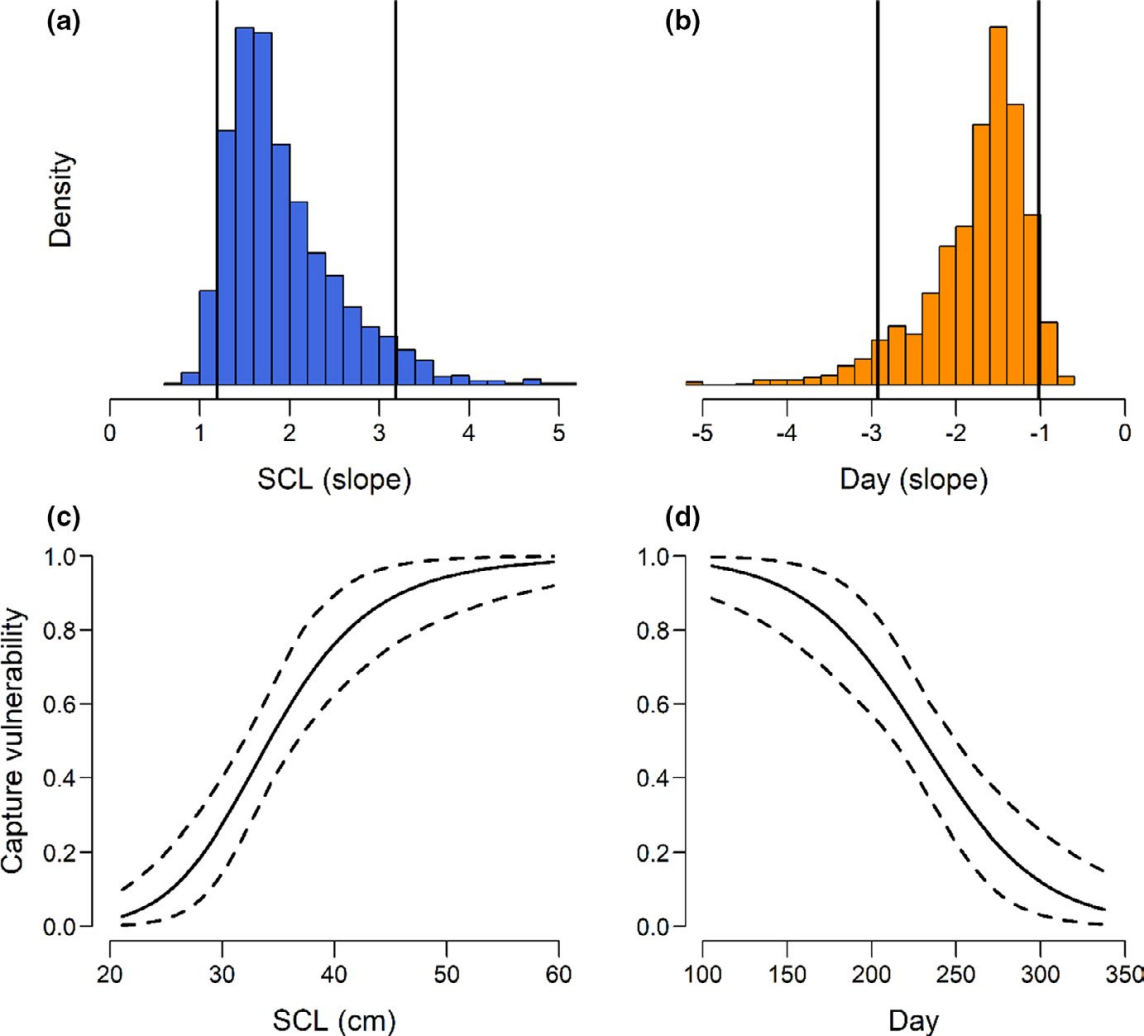
Posterior distribution of the slope with a 90% highest density interval (HDI, vertical lines) for straight carapace length (SCL, panel a) and day of year (day, panel b) and associated pier‐capture vulnerability estimates with 90% confidence limits (dashed lines) across a range of covariate values (panels c and d). The SCL slope and estimates are interpreted as the relationship with pier use with day of year held at mean levels (and vice versa). The curvature in the slopes is an artifact of the scaling to a probability between 0 and 1 from the logit scale and back transformation of SCL from the natural‐log scale. These are straight‐line relationships on the model scale

Green turtle pier‐capture vulnerability was significantly related to both size and time of year, with strong linear relationships (Table 5, Figure 5). Ψ increased significantly with increasing SCL and decreased significantly with increasing day. The HDI for the BCI slope overlapped zero; however, the posterior distribution supported a negative relationship with Ψ (Figure 6). $\hat{R}$ was <1.05 for all model parameters, and trace plots confirmed adequate convergence.

**TABLE 5:** Green turtle pier‐capture vulnerability model estimates

Parameter	Mode (90% HDI)
Intercept	0.24 (−0.27, 0.83)
SCL	*1.76 (1.10, 2.91)
BCI	−0.23 (−0.76, 0.28)
Day	*−1.60 (−2.73, −0.94)
Individual *SD*	0.98 (0.01, 2.92)

Note

Coefficients are reported on the logit scale as the mode (effect size) with a 90% highest density interval (HDI). The intercept is interpreted as estimated capture vulnerability at mean straight carapace length (SCL, 36 cm), body condition index (BCI, 1.29), and day of year (day, August 9). *Highlights covariate slope HDIs that did not overlap zero. Individual standard deviation (*SD*) represents unexplained individual variation in capture vulnerability.

## 4 DISCUSSION

Morphological and behavioral traits are commonly used to assess capture vulnerability of fish in commercial fishing activities (Alós et al., 2014; Klefoth et al., 2017), and results of this study suggest similar forces also affect hook‐and‐line capture of non‐target species such as sea turtles. We identified factors, including size and time of year when individuals are captured, that affect vulnerability of sea turtles to hook‐and‐line capture from a recreational fishing pier in Northwest Florida. Additional characteristics of sea turtles, such as body condition and head width, may also play a role. Individual variability in pier use has also been observed with other marine species including manta rays (*Mobula birostrus*; Pate & Marshall, 2020) and blacktip sharks (*Carcharhinus limbatus*; Spencer, 2017). While direct mortality of these species may not occur in large numbers on fishing piers (Adimey et al., 2014), sublethal wounds and stress from capture and injury may have long‐term, and still unknown, repercussions (Pate & Marshall, 2020).

Kemp’s ridleys are commonly documented as incidental captures on fishing piers (Coleman et al., 2016; Cook et al., 2020; Seney, 2017); however, during our study, green turtles dominated pier captures. Species composition of pier captures most likely reflects the composition and abundance of species using surrounding waters; green turtles are frequently captured in nearshore waters off of northwest Florida (Lamont & Johnson, 2021). Additionally, although the genetic origin of juvenile green turtles captured on the pier is unknown, green turtle nesting has increased exponentially in Florida in recent years (Ceriani et al., 2019) while loggerhead nesting has remained stable (Ceriani et al., 2019) and Kemp’s ridley nesting appears to have declined (Caillouet et al., 2018; Gallaway et al., 2016). Capture of Kemp’s ridleys and loggerheads on recreational fishing piers may not be unexpected considering that the bait typically used by recreational anglers (e.g., fish, shrimp) are known diet items of these species (Molter et al., 2021; Ramirez et al., 2020; Shaver, 1991). However, capture of green turtles on the NAV pier was unexpected as juvenile green turtles in neritic habitats are generally considered herbivores (Williams et al., 2014). Most of the pier‐caught green turtles were foul‐hooked (67%), which may suggest turtles were foraging on algae growing on the pier pilings rather than targeting bait. However, turtles may have also been foul‐hooked while attempting to take bait. Probability of pier capture for green turtles increased with size, which may simply reflect a larger surface area for foul hooking to occur. The six pier‐caught Kemp’s ridleys were also larger than our net‐caught individuals and were also larger than Kemp’s ridleys captured from piers in Mississippi Sound (mean SCL ± *SD*: 36.0 ± 7.5 cm, Coleman et al., 2016).

Exploratory behavior has been linked to body size, and perhaps larger turtles are bolder and more willing to risk foraging around the pier (Darby & McGhee, 2019; Kelleher et al., 2017; Maillet et al., 2015). In our study, green turtles captured both from the pier and in the net were on average 14.5 cm larger when they were pier‐captured (*n* = 11). It is difficult to directly examine boldness in juvenile sea turtles either in a laboratory (Klefoth et al., 2017; but see Kudo et al., 2021) or in the wild (Breck et al., 2019; Hertel et al., 2019). Griffin et al. (2017) were able to observe green turtle behavior in relation to snorkelers; however, in general, sea turtles move large distances, inhabit deep waters, and may remain submerged at depth for several hours. Fine‐scale tracking of individuals has been used to assess boldness, primarily with terrestrial species (Breck et al., 2019; Hertel et al., 2019); however, satellite tags that transmit GPS‐quality locations are not readily available in sizes small enough for juvenile sea turtles. In addition, tracking durations for small turtles are not long enough to assess behavior due to rapid carapace growth (Lamont & Iverson, 2018). Acoustic telemetry using a passive receiver array installed on and in the vicinity of an active fishing pier (such as NAV pier) would provide useful data, particularly when installed as part of a cooperative acoustic network such as iTAG (Friess et al., 2021; Hertel et al., 2019).

Body condition of pier‐caught green turtles and loggerheads did not differ from net‐caught turtles. After being caught on the pier, turtles go to a rehabilitation center where they are examined and treated by a veterinarian (sometimes for weeks or months) before being released, and this care may improve their BCI (Hughes et al., 2019). However, measurements of pier‐caught turtles were collected immediately after capture and, as such, should not have been impacted by rehabilitation efforts, except perhaps for turtles caught multiple times on the pier. We found no difference in BCI between green turtles with multiple pier captures and those captured in the net or captured only once on the pier. Interestingly, BCI of net‐caught green turtles from SRI was lower than BCI of green turtles captured in nearby seagrass habitats of St. Joseph Bay, Florida, approximately 150 km east of SRI. This suggests green turtles in the SRI area are foraging in a less optimal habitat, regardless of whether they are foraging naturally or at the pier (Lamont & Johnson, 2021). Examining stable isotope values for net‐caught and pier‐caught turtles would help clarify the diet of these individuals, including whether pier‐caught turtles are only foraging on bait or also foraging on algae and epibionts growing on the pier structure (Vander Zanden et al., 2013). Our small numbers of recaptured turtles (particularly those with recapture intervals >180 days; Lamont & Johnson, 2021) prohibited us from comparing growth rates of pier‐caught and net‐caught turtles.

As opposed to green turtles, body condition of pier‐caught Kemp’s ridleys was lower than their net‐caught counterparts. However, this may simply reflect variation in body shape as Kemp’s ridley grow. Lamont and Johnson (2021) reported lower BCI values for Kemp’s ridleys >40 cm SCL at SRI as compared to those <40 cm SCL. Mean BCI of our pier‐caught turtles (1.39) was similar to mean BCI reported for 40–49 cm SCL Kemp’s ridleys by Lamont and Johnson (2021; 1.41).

Although there were no statistical differences between head width of net‐caught and pier‐caught turtles, conclusions from those analyses should be taken with caution because of small sample size. Head width is not a standard measurement collected at the rehabilitation facilities; therefore, we were only able to evaluate head width of turtles first captured in the net and then recaptured from the pier (*n* = 5). That being said, head width was lower for individuals captured only in the net (1.69), those captured once on the pier (1.64), and those captured >2 times on the pier (1.586). Head width is a function of bite force (Marshall et al., 2014), and as such, it is reasonable to suspect differences among individuals that forage in different habitats, as has been shown for nesting loggerheads (Price et al., 2017). For example, turtles foraging on algae along rock jetties may have narrower head widths that those foraging in seagrass habitat because seagrass blades are relatively tough and must be torn by the turtle (Marshall et al., 2014). Head width is easy to measure, and we suggest it might be beneficial to add to the suite of data collected from turtles captured from fishing piers.

In addition to body size, pier‐capture vulnerability was also influenced by time of year. Individuals were more likely to be caught on the pier earlier in the year. This corresponds well with angler surveys collected by the National Marine Fisheries Service Marine Recreational Fishery Statistics Survey (MRFSS) that showed shore‐based fishing effort along Florida’s GOM coast was greatest in March–April and lowest in July–August (Braun‐McNeill & Epperly, 2002). We do not have fishing effort data for the NAV pier and were unable to determine whether the seasonal pattern of turtle captures documented on the pier was a function of fishing effort or turtle behavior (or both). A similar pattern was observed in both Apalachicola Bay, Florida, and on piers in Galveston County, Texas, where approximately 50% of hook‐and‐line captures occurred in May and June (Rudloe & Rudloe, 2005; Seney, 2017). However, anglers on piers reported capturing most turtles between June and August in Mississippi (Cook et al., 2020). Seasonal variation in pier captures could also reflect movements of juvenile turtles into neritic, summer foraging areas from deep‐water, wintering home ranges (Lamont & Iverson, 2018; Metz et al., 2020). This type of movement was not reflected in our net captures, however.

Although we identified characteristics that differed between pier‐caught and net‐caught turtles, our results suggest that the capture vulnerability of sea turtles at fishing piers may be driven by a relatively complex suite of factors. Although it was not significant in our dataset, the results support a potential negative relationship between BCI and capture vulnerability with size held constant. Thus, larger turtles with lower body condition may be more likely to be pier‐captured than similar‐sized individuals with higher body condition. The complexity in relationships between turtle behavior, morphology, and life‐history traits appears to exist in the factors that contribute to capture vulnerability of sea turtles at fishing piers.

### 4.1 Conservation implications

It has been suggested that mortality in recreational fishing activities is resulting in selection against specific morphological traits, such as body depth and mouth size in target fishes (Alós et al., 2014). Mortality of sea turtles captured from fishing piers could be having a similar impact on sea turtle populations, albeit over a much longer time scale. Additionally, sublethal injuries and stress from capture and handling may have population‐level impacts. In a 6‐year period (2010–2015), more than 1000 sea turtles were captured by recreational anglers in Mississippi alone (Cook et al., 2020). Capture in recreational fishing activities may result in selection against bolder turtles and that personality trait could impact the overall population, not just pier‐captured turtles, by increasing exposure to threats such as predators (Griffin et al., 2017) or altering a turtle’s ability to adjust to changing temperatures (Clark et al., 2017; Pich et al., 2019). On the other hand, bolder turtles may be more exploratory and that could benefit range expansion in populations as they adapt to a changing climate (Osland et al., 2021). Populations that contain a mix of behavior types may be most resilient to anthropogenic and natural pressures that affect these species over time (Griffin et al., 2017; Schindler et al., 2010).

## CONFLICT OF INTEREST

The authors declare no conflicts of interest. Any use of trade, product, or firm names is for descriptive purposes only and does not imply endorsement by the U.S. government.

## AUTHOR CONTRIBUTIONS

**Margaret M. Lamont:** Conceptualization (lead); data curation (lead); formal analysis (supporting); funding acquisition (lead); investigation (lead); methodology (equal); project administration (lead); writing – original draft (lead); writing – review & editing (equal). **Robert Mollenhauer:** Conceptualization (supporting); data curation (supporting); formal analysis (lead); writing – original draft (supporting); writing – review & editing (equal). **Allen M. Foley:** Data curation (supporting); project administration (supporting); writing – original draft (supporting); writing – review & editing (equal).

## DISCLOSURE

Any use of trade, product, or firm names is for descriptive purposes only and does not imply endorsement by the U.S. government. This draft manuscript is distributed solely for purposes of scientific peer review. Its content is deliberative and pre‐decisional, so it must not be disclosed or released by reviewers. Because the manuscript has not yet been approved for publication by the U.S. Geological Survey (USGS), it does not represent any official USGS finding or policy.

## Data Availability

The datasets generated for this study will not be made publicly available. Restrictions apply to the datasets. Raw data are exempt from publication due to the sensitivity of endangered species information. Requests to access the datasets should be directed to the corresponding author. All other data used for analyses are presented in the manuscript.
